# Induction of Specific Immunotherapy with Hymenoptera Venoms Using Ultrarush Regimen in Children: Safety and Tolerance

**DOI:** 10.1155/2012/790910

**Published:** 2011-07-19

**Authors:** Alice Köhli-Wiesner, Lisbeth Stahlberger, Christian Bieli, Tamar Stricker, Roger Lauener

**Affiliations:** ^1^Christine Kühne-Center for Allergy Research and Education, University Children's Hospital Zurich, Steinwiesstraße 75, 8032 Zurich, Switzerland; ^2^Children's Allergy and Asthma Hospital, Hochgebirgsklinik Davos, 7265 Davos, Switzerland

## Abstract

*Background & Objective.* Ultrarush induction for specific venom immunotherapy has been shown to be reliable and efficacious in adults. In this study its safety and tolerance in children was evaluated. *Methods.* Retrospective analysis of 102 ultrarush desensitizations carried out between 1997 and 2005 in 94 children, aged 4 to 15 years. Diagnosis and selection for immunotherapy were according to recommendations of the European Academy of Allergy and Clinical Immunology. Systemic adverse reactions (SARs) were described using the classification of H. L. Mueller. *Results.* All patients reached the cumulative dose of 111.1 **μ**g hymenoptera venom within 210 minutes. Six patients (6%) had allergic reactions grade I; 2 patients (2%) grade II and 5 patients (5%) grade III. Three patients (3%) showed unclassified reactions. SARs did not occur in the 15 patients aged 4 to 8 years and they were significantly more frequent in girls (29%) compared with boys (12%) (*P* = 0.034, multivariant analysis) and in bee venom extract treated patients (20%) compared to those treated with wasp venom extract (8%) (OR 0.33, 95% Cl 0.07–1.25). *Conclusion.* Initiation of specific immunotherapy by ultrarush regimen is safe and well tolerated in children and should be considered for treating children with allergy to hymenoptera venom.

## 1. Introduction

Hypersensitivity to hymenoptera venom affects approximately 1%–5% of the general population. In Switzerland the prevalence is estimated to be 3.5% in the general population and 0.4%–0.8% in children aged 4 to 16 years. It is one of the three most common causes of immediate type anaphylactic reactions, the other two major causes being drugs and foods [1]. A field sting in a hymenoptera venom allergic patient can cause a spectrum of reactions ranging from severe local swelling to anaphylactic shock with circulatory collapse. Several cases of death are attributed to hymenoptera allergy yearly, mostly in adults. 

Specific immunotherapy (SIT) is the only known causal treatment for venom-allergic patients [2, 3]. In subjects with a history of generalized reactions to insect sting SIT results in up to 95% rate of protection in wasp venom allergic patients and 80% in bee venom allergic patients [4]. Different protocols have been published for the stepwise increase of the dose of the insect venom during initiation of subcutaneous specific immunotherapy [5–9]: the conventional regime, with injections using increasing doses every one to two weeks over a period of 2 to 4 months, rush immunotherapy extending over approximately 1 week and ultrarush protocols, in which the maintenance dose is achieved in 1-2 days. The latter has been shown to be effective and time-saving in adults [5]. The aim of this retrospective study was to investigate the safety and tolerance of ultrarush induction in desensitization in children. 

A systemic grading of reactions to immunotherapy is necessary in order to evaluate the safety of the treatment and in order to compare various regimens [10]. In this study we chose to use a modified version of the classification of generalized allergic reactions introduced by H. L. Mueller in 1966 [11]. This classification is presented in Table 2 for quick reference.

## 2. Methods

### 2.1. Patients

Medical records of 94 children treated with the ultrarush induction regime in the intensive care unit of the University Children's Hospital of Zurich between January 1997 and December 2005 were analysed retrospectively; the clinical data is summarized in Table 1. Indication for SIT was a combination of an immediate systemic allergic reaction grade III or IV after a hymenoptera field sting and detection of specific IgE antibodies to the venom, as recommended by the European Academy of Allergy and Immunology Subcommittee on insect venom allergy [4, 6]. Patients with less severe reactions were included when the risk of exposure was very high, for example, when a wasp nest was in close proximity to the home, or when the fear from getting a sting caused anxiety and a significant limitation in the quality of life.

The study population included 24 girls and 70 boys; 61 of the patients were allergic to bee venom, 33 to wasp-venom; and 8 boys to both. These 8 boys were considered twice in the evaluation. The patients were divided into three groups according to age: group A included 15 patients aged 4 to 8 years, in group B there were 60 subjects aged 8 to 12 years and in group C 27 patients aged 12 to 15 years. The classification of generalized allergic reactions according to H. L. Mueller which was used to define the adverse reactions is shown in Table 2.

### 2.2. Tests

Sensitization was detected by skin prick tests with 10, 100, and 300 *μ*g/mL purified insect venom extract (Pharmalgen, ALK) and/or intradermal tests with 0.00001, 0.001, 0.01, 0.1, and 1.0 *μ*g/mL purified venom extract (ALK-SQ, ALK-Scherax, Germany). The tests were considered positive if a weal of at least 3 mm in diameter occurred after 15 minutes, and, in the case of intradermal tests, a reaction was considered positive at a concentration of 1 *μ*g/mL or less. Positive (1% histamine hydrochloride) and negative (sodium-chloride 0.9%) control tests were performed. In addition levels of specific IgE were determined in serum (Pharmacia ImmunoCAP System, Sweden); they were considered negative when less than 0.35 kU/L.

### 2.3. Induction of Specific Immunotherapy Using the UltraRush Regime

**Figure 1:**
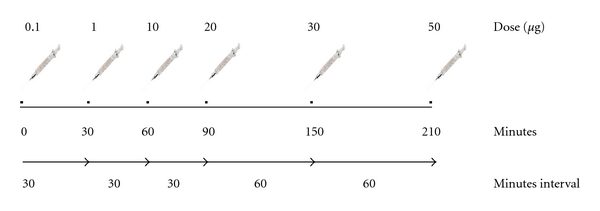
Ultrarush induction regimen in desensitization with subcutaneous venom injections.

The ultrarush induction regime is described in Figure 1. All patients were treated with a standardized purified venom preparation (ALK Pharmalgen, Trimedal) given in subcutaneous injections. The cumulative dose of 111.1 *μ*g hymenoptera venom was reached after 210 minutes using at least 6 injections. In the case of side effects, the interval between the doses was extended. The patients were observed in the intensive care unit with intravenous access, continuous measurements of the oxygen saturation, and repeated measurements of the blood pressure. Three hours after the last injection the patients were discharged to home. Booster injections were given later as follows: on day 7 two doses of 50 *μ*g (ALK Pharmalgen, Trimedal) were given with an interval of 30 minutes, and 3 and 7 weeks after the ultrarush procedure the patients received 100 *μ*g of Alutard (Aluminiumhydroxid-depot-preparation, ALK) subcutaneously. During the entire therapy the children did not receive premedication with antihistamines.

If both wasp and bee venoms sensitizations were required; they were administered in separate protocols a few days apart.

## 3. Results

Between January 1997 and December 2005 94 children, aged 4 to 15.1 years, including 70 males and 24 females, underwent induction of specific immunotherapy using ultrarush regime in the intensive or intermediate care unit of the University Children's Hospital of Zurich. A total of 102 ultrarush immunotherapy induction procedures were performed, in all of which the cumulative dose of 111.1 *μ*g was reached. 61 patients were treated with bee venom, 33 with wasp venom, and 8 boys with both. Average duration of the procedure was 3.5 hours with a range of 2.5 to 5.5 hours.

### 3.1. Adverse Reactions

All patients had local swelling and redness of the upper arm with a diameter of less than 10 cm. As summarized in Table 3, Systemic side effects were observed in 16 subjects (16%), 11 of them required treatment. 6 patients (6%) showed an allergic reaction grade I, 2 girls (2%) grade II, and 5 patients (5%) developed a grade III reaction, 1 of these 5 subjects recovered spontaneously. No grade IV reactions occurred. 3 (3%) patients showed a reaction which could not be classified according to Mueller, namely, prickle of the tongue and throat and dizziness. Severe adverse reactions occurred mainly after injection of 50 *μ*g venom (9 subjects) and more often in bee venom (13 patients, 20%) than in wasp venom (3 patients, 8%) allergic subjects; this latter tendency however, was not statistically significant (OR 0.33, 95% Cl 0.07–1.25, *P* = 0.0955).

 Overall, 29% of the girls, compared to only 12% of the boys, developed a systemic adverse event; this difference was statistically significant (*P* = 0.034, multivariant analysis). None of the patients in group A (4 to 8 years of age) showed systemic side effects compared to 18% in group B (8 to 12 years of age) and 19% in group C (12 to 15 years of age). All but one reaction occurred within 30 minutes after injection of venom. In one boy generalised urticaria developed 3 hours after injection.

## 4. Discussion

When starting specific immunotherapy, various protocols for increasing the dose of allergen up to the maintenance dose have been introduced in the past years, attempting to maximize protection, minimize side effects, and optimize patient convenience. It is difficult to compare the results because the regimens differ. Increasing data in adults demonstrate good tolerance and safety for the ultrarush induction in insect venom immunotherapy. For example, Birnbaum et al. found fewer systemic reactions with a 3.5-hour (210 min) protocol with a cumulative dose of 101.1 *μ*g venom compared to 6-hour and to 4-day protocols, which attained cumulative doses of 226.6 *μ*g and 527.6 *μ*g, respectively [5, 7]. The nine years of experience with initiation of specific immunotherapy to insect venom by ultrarush protocol in paediatric subjects, which is summarized in this paper, demonstrates that ultrarush insect venom immunotherapy is a well-tolerated and safe induction regimen also in children. Few side effects were observed, no cardiac or circulatory side effects and no systemic allergic reactions in the youngest age group (4–8 years). Three subjects had unclassified reactions with prickle of the tongue and throat and dizziness. These reactions did not fit the usual categories of allergic reactions, and it was not clear whether they were related directly to the immunotherapy or whether they were caused by the circumstances of the treatment (hospitalization in the intensive/intermediate care unit, subcutaneous injections, monitoring, etc.). In order to assess this question a control group undergoing the same procedures but getting placebo injections would be needed. However, this was beyond the scope of this retrospective study. 

Antihistamines are efficiently and widely used to suppress allergic symptoms. There are studies which support the strategy of premedication with antihistamines in order to reduce allergic side effects and enhance the safety and efficacy of allergen-specific immunotherapy [12, 13]. However, there is also data which suggests that medication with antihistamines may impair allergen-specific immunotherapy [14]. The patients in this retrospective study did not receive treatment with antihistamines prior to immunotherapy. 

The results presented in this paper compare favourably with the frequency of side effects reported in adults and with the incidence of severe adverse reactions in conventional and rush protocols [5, 7, 15–20]. The ultrarush induction protocol performed in a paediatric intensive or intermediate care unit allows for much better monitoring of the patients compared to the conventional desensitization protocol performed in an outpatient setting. Its short duration is much more convenient for the patients and their parents, and it has the additional advantages of achieving rapid protection as well as reduction in costs. Based on the results presented in this paper and due to these considerations, we suggest that the ultrarush induction regimen for desensitization, when performed in a suitable intensive care setting, will be considered the treatment of choice in paediatric patients with hymenoptera venom allergy who qualify for immunotherapy.

## Figures and Tables

**Table 1:** Clinical data of children undergoing ultrarush venom immunotherapy.

No. patients	Total 94	Bee venom ultrarush	Wasp venom ultrarush
Age in years			
Range	4.0–15.1		
Mean, median	10.4, 10.5		

Gender			
Boys	78 (76.5 %)	47 (46.1%)	31 (30.4%)
Girls	24 (23.5 %)	18 (17.6%)	6 (5.9%)

Allergen			
Bee venom	57 (55.9 %)	65 (63.7%)	
Wasp venom	29 (28.4 %)		37 (36.3 %)
Both	8 (7.8 %)		

Grade of reaction to field sting			
I	1 (1.0%)	1	0
II	20 (19.6%)	13	7
III	60 (58.8%)	37	23
IV	15 (14.7%)	9	6
Other (Sensitisation)	6 (5.9%)	5	1

**Table 2:** Classification of allergic reactions after HL Mueller, modified.

	Reaction
Large local reaction	Swelling at site of sting with diameter >10 cm, lasting >24 h

Grade I	Generalized urticaria, itching, malaise, anxiety

Grade II	Any of the above, plus two or more of the following: angiooedema (grade II also if alone), constriction in chest, nausea, vomiting, diarrhoea, abdominal pain, dizziness

Grade III	Any of the above, plus two or more of the following: dyspnoea, wheezing, stridor (any of these alone are grade III), dysphagia, dysarthria, hoarseness, weakness, confusion, fear of death

Grade IV	Any of the above, plus two or more of the following: drop of blood pressure, collapse, loss of consciousness, incontinence (urine, stool), cyanosis

**Table 3:** Subjects with side effects.

Sex	Age (y)	Insect	Grade at sting	Side effects	Grade	At dose in *μ*g	Therapy
F	8.1	Bee	II	*Generalized urticaria, cough *(no wheezing, no dyspnoea, no stridor)	II	50	Antihistamines, Corticosteroids i.v.
M	9.5	Bee	Sensit. (IV)	*Dizziness*	other	30	None
M	9.5	Bee	II-III	*Generalyzed urticaria*	I	30	Antihistamines i.v.
M	10.9	Bee	III	*Urticaria, dyspnoea, wheezing *	III	30	Antihistamines, Corticosteroids i.v., Salbutamol-inhalation
M	14.7	Bee	III	*Generalized urticaria; dyspnoea, constriction in chest*	IIII	2050	Antihistamines, Corticosteroids i.v.dito + inhal. of Adrenalin/Salbutamol
F	10.3	Bee	II-III	*Urticaria* about 3 h after last injection (50 *μ*g)	I	50	Antihistamines, Corticosteroids i.v.
F	10.4	Bee	II	Slight *dyspnoea*; fast, spontaneous normalization	III	1	None
M	12.1	Bee	III-IV	*Generalized urticaria*, itching in the throat	I	30	Antihistamines, Corticosteroids i.v
M	10.5	Bee	II-III	*Itching in the throat *	other	50	Cetirizin per os
F	8.7	Bee	III	*Chest pain, inspiratory stridor, chest pain, in-and expiratory stridor*	IIIIII	1050	Antihistamines, Corticosteroids i.v., Adrenalin-inhalation dito + Salbutamol-inhalation, Corticosteroids i.v. (before going home)
F	14.1	Bee	III	*Slight periorbital swelling *	II	1, 30 and 50	Antihistamines i.v. after 1ug
M	15.1	Bee	II	Itching in meatus acusticus, *rash* chest, *several urticarial lesions* on the left arm	I	50	None
F	15.0	Wasp	III	*Dysphagia, passing dyspnea *	III	0.1	Antihistamines and Corticosteroids i.v, and again before going home
F	11.3	Wasp	III	*Slight prickle of the tongue*	other	10	None
M	10.3	Bee	IV	Redness and *one urticarial lesion on the left cheek *	I	50	none, 1 Levocetirizine per os before going home
M	10.7	Wasp	III	*Generalyzed urticaria *	I	50	Levocetirizin per os
All children				Local redness and swelling, sometimes overheating and itching at the injection site. Therapy if needed: Coldpack and/or Antihistamin gel			
